# Artifacts Mimicking Hardware Loosening After Iterative Metal Artifact Reduction (iMAR) Processing in Spinal Surgery

**DOI:** 10.7759/cureus.77766

**Published:** 2025-01-21

**Authors:** Jason P Park, Anton O Beitia, Avraham Y Bluestone, Vishal K Patel, Patricia E Roche

**Affiliations:** 1 Radiology, Stony Brook University Hospital, Stony Brook, USA; 2 Radiology, Neuroradiology, Stony Brook University Hospital, Stony Brook, USA

**Keywords:** computed tomography, metal artifact reduction, postoperative imaging, pseudolucency, spinal fusion

## Abstract

Metallic implants often produce artifacts in computed tomography (CT) imaging, complicating the interpretation of postoperative findings. This case report describes a 63-year-old male with a history of cervical and lumbar spine surgeries who presented with worsening radicular pain. The patient underwent cervical decompression laminectomy and posterior instrumented fusion. Postoperative CT, processed with an iterative metal artifact reduction (iMAR) algorithm, revealed unexpected hypodense lucencies surrounding pedicle screws and fixation hardware, initially suggestive of hardware loosening or infection. However, these lucencies were not seen in non-processed images, indicating their artifactual nature. This case highlights the potential for iMAR to generate misleading findings that may mimic clinical conditions, emphasizing the need for a careful approach when interpreting iMAR-processed images in conjunction with clinical context and pre-processed datasets to prevent unnecessary interventions.

## Introduction

Metallic implants are known to cause significant artifacts on computed tomography (CT) that degrade image quality through various mechanisms such as beam hardening and photon starvation. Recently, many post-processing algorithmic methods have been developed to reduce these artifacts without increasing radiation dose. These include projection-based iterative metal artifact reduction (iMAR) algorithms which are commercially available through different vendors. However, these algorithms are not perfect and while minimizing artifacts from metallic implants, they may introduce less widely recognized artifacts of their own. In particular, we evaluate a case of an iMAR algorithm artifact presenting as periprosthetic radiolucency mimicking the loosening of cervical spinal fusion hardware.

## Case presentation

We present a 63-year-old man with a past medical history of cervical and lumbar spine stenosis status post-C3-C5 anterior cervical discectomy and fusion (ACDF) 30 years ago and posterior spinal fusion of L4-S1. The patient presented to the orthopedic office with worsening pain radiating to both shoulders and lower extremities. Preoperative MRI and CT demonstrated moderate C2-C3, severe C5-C6, and mild to moderate C6-T2 central spinal stenosis suggestive of adjacent segment disease. A decision was made to proceed with cervical spinal decompression C5-C7 laminectomies and posterior instrumented spinal fusion from C2-T2.

The patient’s surgery was technically successful without immediate post-surgical complications. The patient had an uncomplicated hospital course and was discharged on the third postoperative day without any motor or sensory deficits and with pain well controlled on oral analgesics.

CT of the cervical spine was performed on the first day following surgery using 1.25 mm thick helical axial slices with coronal and sagittal reconstruction without and with iterative MAR post-processing (iMAR; Siemens Healthineers, Forchheim, Germany). Initial review of the pre-processed images demonstrated significant streak artifacts extending from previously placed ACDF anterior fixation hardware and dental hardware. After post-processing, there was a marked reduction in these streak artifacts, including those from dental hardware not included in the field of view. Furthermore, there was preservation of morphology and details of the hardware, including the crevices of the spinal rod and pedicle screw interface.

**Figure 1:**
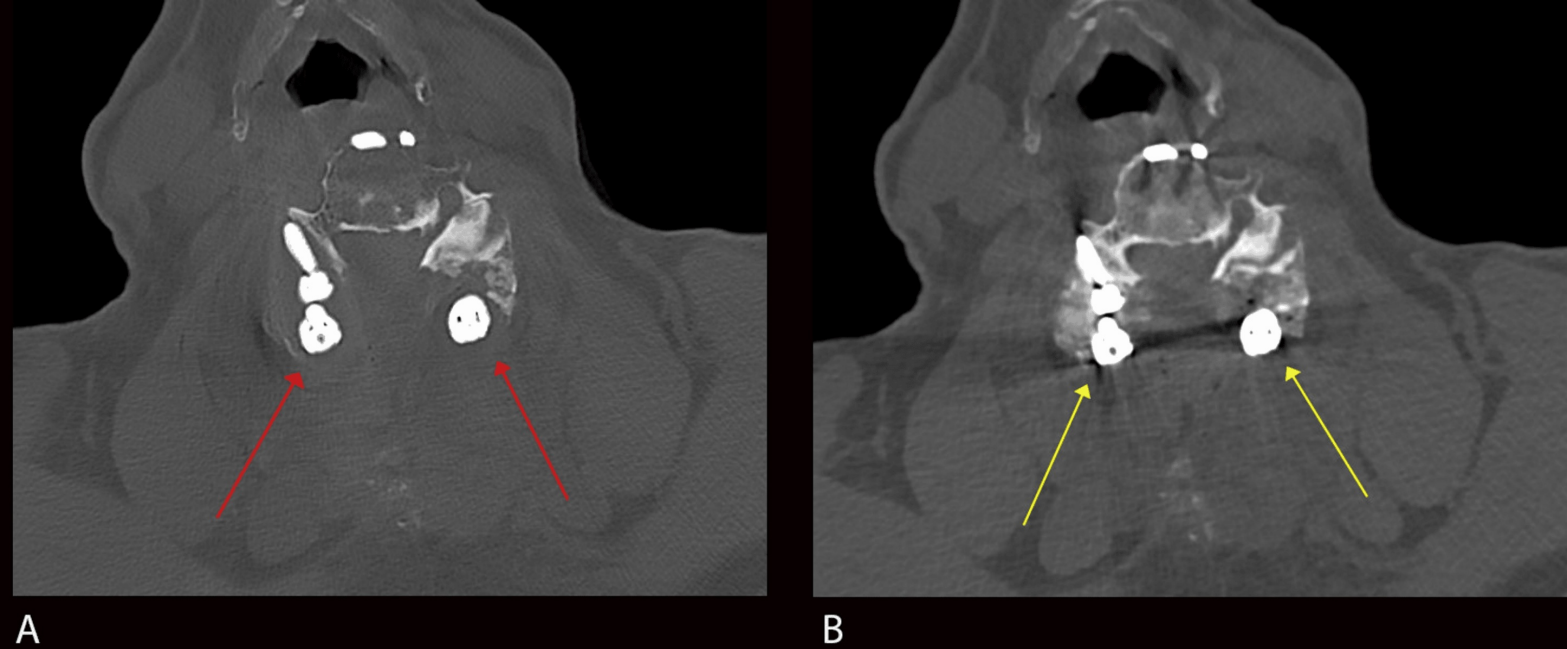
Axial image of the cervical spine with iMAR post-processing (A) and without iMAR post-processing (B) Figure 1A shows lucencies surrounding the pedicle screws at the C6 level (red arrows), mimicking findings associated with hardware loosening. These do not persist on axial CT images without iMAR post-processing (yellow arrows in Figure 1B) and the normal interface between the pedicle screws and osseous structures is shown. These findings are consistent with iMAR-related artifacts. iMAR: iterative metal artifact reduction

**Figure 2:**
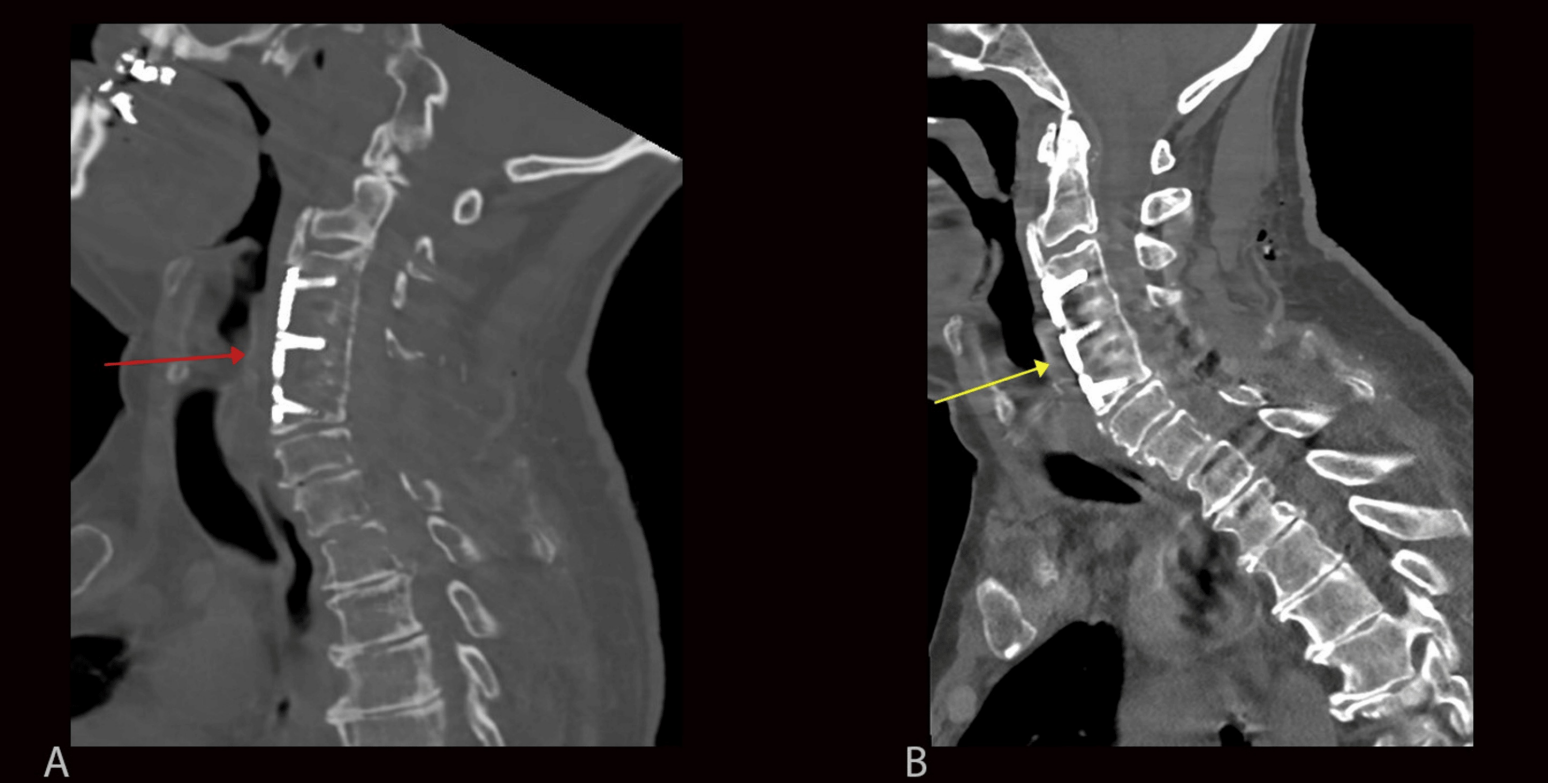
Sagittal images of the cervical spine with iMAR post-processing (A) and without iMAR post-processing (B) Figure 2A shows an iMAR post-processed image with lucencies surrounding the anterior cervical spine fixation hardware (red arrow). These lucencies do not persist on sagittal CT images without iMAR post-processing (yellow arrow, Figure 2B) but rather demonstrate normal trabecular bone with fairly minimal overlying streak artifact. These findings suggest the absence of hardware loosening and are consistent with iMAR-generated artifacts. iMAR: iterative metal artifact reduction

In addition to expected post-surgical changes, the iMAR-processed images demonstrated hypodense lucencies surrounding the lateral mass screws at multiple cervical spine levels (Figure 1A). The lucencies measured approximately up to 3-4 mm orthogonal to the pedicle screws, and density measurements of approximately 60-120 Hounsfield units. Additional lucencies were seen surrounding the previously placed ACDF hardware (Figure 2A). The differential diagnosis for the periprosthetic lucencies included artifact or hardware loosening. Given the immediate postoperative nature, additional differential diagnoses of acute infection or severe osteoporotic bone were also considered. However, these periprosthetic lucencies were not visualized on the images without iMAR post-processing (Figures 1B-2B). Therefore, these lucencies were thought to represent iMAR-generated artifacts.

## Discussion

Periprosthetic loosening is a common form of hardware failure after spinal fusion surgery. The incidence ranges from 0.8% to 27%, with significantly higher rates occurring in those with osteoporosis. Hardware loosening may occur from excessive mechanical forces, such as shear forces between the spinal screws and bone resulting from muscle contraction and spine movement. Likewise, contamination of hardware can lead to infection and biofilm formation, which has been associated with osteolysis. Treatment may involve a combination of antibiotics, replacement of the screws, and refusion of the spine. In either case, accurate imaging evaluation for periprosthetic hardware loosening is an essential part of determining appropriate hardware placement or the need for revision [1].

CT criteria for periprosthetic loosening include 2 mm or greater surrounding the hardware, particularly when this lucency enlarges on sequential studies [2]. An additional radiographic sign suggesting loosening is the “double halo” sign, defined as the presence of a radiolucent area and radiopaque rim on the same radiograph [3]. Although the “double-halo” was described as a radiographic sign, a CT correlate is often seen in cases of loosening.

Various methods have been developed to correct for artifacts related to metallic implants of which iMAR is a leading method used in clinical practice. A brief summary of the iMAR technique follows. More detailed descriptions of the iMAR technique can be found in the references [4-6]. In CT, a photon detector is rotated around the patient at varying angles to obtain a sinogram, a data array storing projection values of an object at varying angles. Projection-based iMAR algorithms begin with the segmentation of the metallic hardware and associated artifacts through methods such as Hounsfield thresholding. These regions are removed from the original unsegmented sinogram and replaced with interpolated data from surrounding tissue, a process known as sinogram inpainting. The corrected sinogram is then back-projected to form an image with reduced metal artifacts. This process can be iteratively repeated until a threshold is met, resulting in a final corrected image [4-6].

While these methods have made significant advances in the reduction of streak artifacts, newer artifacts have emerged, a few of which may be confused with clinical pathology. In our case, iMAR-processed images demonstrated lucencies surrounding the spinal fixation hardware, mimicking findings associated with hardware loosening. As previously stated, periprosthetic hardware loosening is diagnosed when the lucencies exceed two millimeters. Our images exhibited lucencies measuring approximately 3-4 mm, meeting this criterion. However, the lucencies that are usually confined to the lateral mass screws were visualized throughout the entirety of the fixation hardware, including the anterior fixation plate, posterior rods, and screw heads (Figures 1A and Figure 2A). Likewise, a double halo sign that is often seen with pedicle screw loosening was absent in our iMAR-processed images. Additionally, on iMAR post-processed images, there was a generalized decrease in bone density, even at the levels where there is no implant. This finding, which was not present on the pre-processed images, also hints against the diagnosis of implant loosening. Other clinical features, such as the uncomplicated immediate postoperative period and lack of clinical signs of infection, made hardware loosening less likely. Another point is that the exam was performed one day post-surgery, and for implant loosening to occur at this time would be very unusual. Most importantly, careful examination of regions not obscured by streak artifacts on pre-processed images demonstrated normal apposition of bone and hardware without separation (Figure 1B and Figure 2B). This absence of periprosthetic lucencies on pre-processed images, as well as the other aforementioned clinical and imaging findings, strongly supported the artifactual nature of the finding.

Similar artifacts have been previously described in the literature. One study by Neroladaki et al. demonstrated similar findings of pseudo-loosening in their evaluation of iMAR-processed images of patients with hip prostheses [5]. While 28 patients were evaluated, it is unclear the number of scans that demonstrated this artifact. Similarly, Hakim et al. frequently identified an inner hypodense and outer hyperdense rim surrounding brain aneurysms treated with vascular coils [7]. However, this was not confused with any type of clinical pathology.

Other iMAR-related artifacts include blooming artifacts and the creation of new streak artifacts. Do et al. described the creation of blooming artifacts around the antenna during microwave ablation experiments in pigs [8]. Similarly, Wuest et al. demonstrated a significant reduction in streak artifacts from dental hardware after post-processing, but with the creation of new streak artifacts in remote areas such as the spinal cord. This occurred in approximately nine out of 50 patients (18%) [9]. These artifacts were not readily evident in our patient’s postoperative imaging.

Since the emergence of these iMAR-related artifacts, additional modifications have been developed, such as normalized metal artifact reduction (NMAR) and frequency split metal artifact reduction (FSMAR). In NMAR, it is theorized that streak artifacts originate from inhomogeneous data and lack of smoothness between the interpolated and original data. Normalization of the original sinogram prior to interpolation leads to increased data homogeneity and reduction of streak artifacts [10]. On the other hand, FSMAR attempts to minimize excessive information loss during deletion and replacement of corrupted data from the inpainting sinogram method. FSMAR aims to preserve some of the edge information by computing a weighted sum of a low-pass filtered MAR image with a combination of a high-pass filtered source and MAR images [11]. FSMAR may be useful in minimizing the pseudolucencies seen in our patient, which likely stems from excessive information loss and inaccurate interpolation at the bone metal interface. Siemens iMAR incorporates both NMAR and FSMAR in its metal reduction algorithm [6].

## Conclusions

The reduction of artifacts from metallic hardware is accomplished well through recently developed iMAR post-processing algorithms. However, these algorithms may introduce new artifacts of their own partly due to excessive data loss and imperfect interpolation. To our knowledge, this is the first published case demonstrating artifactual loosening of spinal fixation hardware after iMAR post-processing. This pseudo-loosening has many clinical implications that may subject patients to unnecessary interventions when interpreted incorrectly. Interpretation of iMAR-processed images therefore remains to be performed with pre-processed source images and available clinical information.
